# The inhibitory activities of two compounds from *Securidaca longepedunculata* Fresen on the acetylcholinesterase from wheat pest *Schizaphis graminum* Rondani: *in silico* analysis

**DOI:** 10.1080/15592324.2024.2444311

**Published:** 2024-12-19

**Authors:** Rasmané Guiré, Pousbila Salo, Eliasse Zongo, Mohamed Fawzy Ramadan, Benjamin Kouliga Koama, Roland Nag-Tiero Meda, Fahad Al-Asmari, Muhammad Abdul Rahim

**Affiliations:** a Laboratory of Research and Teaching in Animal Health and Biotechnology, Universite Nazi Boni, Bobo-Dioulasso, Burkina Faso; b Department of Clinical Nutrition, Faculty of Applied Medical Sciences, Umm Al-Qura University, Makkah, Saudi Arabia; c Department of Food and Nutrition Sciences, College of Agricultural and Food Sciences, King Faisal University, Al-Ahsa, Saudi Arabia; d Department of Food Science & Nutrition, Faculty of Medicine and Allied Health Sciences, Times Institute, Multan, Pakistan

**Keywords:** Acetylcholinesterase, *S. longepedunculata*, *S. graminum*, molecular docking, molecular dynamic simulation

## Abstract

Wheat is the third most widely consumed cereal in the world, after maize and rice. However, it is regularly attacked by the wheat aphid (*Schizaphis graminum*), causing considerable damage to wheat crops. The acetylcholinesterase enzyme, which plays a key role in the transmission of the synaptic cholinergic signal, has emerged as a promising target for the development of pest control strategies. Inhibition of this enzyme leads to the paralysis or even death of the aphid. The objective of this study is to identify the bioactive compounds in *Securidaca longepedunculata (S. longepedunculata)* that are capable of interacting with acetylcholinesterase from *Schizaphis graminum* and inhibiting its activity. Furthermore, a computer simulation of these compounds in interaction with the key protein was conducted. First, the secondary metabolites of *S. longepedunculata* were selected on the basis of GC-MS data available from specific reference sources. Subsequently, the compounds were subjected to virtual screening based on their docking scores in order to identify those with inhibitory properties. The compounds with the highest scores were subjected to molecular dynamics simulation over a 50 ns trajectory. Subsequently, MMGBSA free energy calculations were conducted. The results demonstrated that eight compounds exhibited inhibitory properties, four of which (echimidine, populin, salidroside, and farrerol) demonstrated superior stabilizing effects on proteins compared to the remaining compounds. In terms of free energy by MMGBSA and molecular simulation, it was observed that echimidine and populin formed robust and stable hydrogen bonds with the amino acids of the acetylcholinesterase enzyme. This study identifies and attempts to validate the potential inhibitory activities of echimidine and populin against acetylcholinesterase, with a view to developing potent insecticides and unique treatment strategies.

## 1. Introduction

The issue of pest management has become a prominent concern in the pursuit of food security for all. Aphids, one of the most prevalent pests, have been estimated to cause significant damage to 15 million hectares of crops, representing a significant loss.^1^ It is unfortunate that the majority of control methods are based on synthetic pesticides, which are beginning to demonstrate their limitations as their use increases due to the hormesis phenomenon.^2^

The principal effect of the various pesticides is on the insects’ central nervous system. Aphids employ eight biochemical or molecular energetic mechanisms^3^ to evade the insecticide effect. The most active mechanisms primarily involve modifying the active site or structure of the enzyme, the overproduction of metabolizing enzymes, and the increased excretion or reduced penetration of the insecticide.^4^

Acetylcholine is a vital neurotransmitter in the transmission of chemical signals at cholinergic receptors in the central nervous system of insects. The enzyme acetylcholinesterase is essential for the optimal functioning of cholinergic synapses and plays a pivotal role in this process. Upon binding to cholinergic receptors, acetylcholine facilitates the opening of sodium and potassium channels, thereby generating a post-synaptic excitatory potential.^5^ The emission of this post-synaptic excitatory potential is terminated when acetylcholine is hydrolyzed by acetylcholinesterase, resulting in the closure of sodium and potassium channels. In the absence of acetylcholinesterase, the accumulation of acetylcholine in synapses results in the hyperexcitation of cholinergic receptors, which ultimately leads to insect death.

The regulation or inhibition of acetylcholinesterase represents a pivotal factor in the life cycle of aphids. Specific pesticides, including carbamates and organophosphates, have been demonstrated to inhibit acetylcholinesterase activity.

To evade the impact of these pesticides, a particular group of insects undergoes a specific genetic alteration,^6^ which involves multiple genes with intricate genetic interactions.^7^ The resistance is more pronounced when expressed in the homozygous state, resulting in a decline of over 60% in enzyme activity.^8^ Each mutation is defined for a specific insecticide category.^9^ The process of detoxification is an essential component of an insect’s survival strategy, enabling them to protect themselves from xenobiotics.^10^ The limited differences between insect antixenosis and antibiosis phenomena^11^ lead us to believe that further investigation is required. Natural resources, including plants, constitute a reservoir of secondary metabolites that can be exploited for the development of new drugs.^12^ The findings of^13^ indicated that phytocompounds, including echihumiline, echihumiline N-oxide, 3‘acetylheliosupine, seneciphylline, and heliosupine, demonstrated a notable inhibitory effect on acetylcholinesterase.

To illustrate, *Securidaca longepedunculata* (*S. longepedunculata*, Polygalaceae) is a rich source of a diverse array of highly active secondary metabolites with a broad spectrum of biological activities, including insecticidal properties. One of the main constituents of *S. longepedunculata*, namely polyphenols, particularly flavonoids, is instrumental in neutralizing aphids. In order to circumvent this action, insects activate their enzymatic systems by hydrolyzing^14^ or oligomerizing^15^ flavonoid compounds by acylation,^16^ thereby converting them from hydrophobic to hydrophilic, water-soluble, and unstable compounds.

Enzymatic processes represent a promising avenue for restoring or even enhancing the stability and properties of flavonoids. The selective synthesis of acylated derivatives is made possible by enzymes, which could prove invaluable in this regard.^17^ This approach ensures that the hydroxyl groups responsible for the desired biological effects remain intact. The utilization of biologically active, target-specific compounds, such as enzymes or receptors, to control insects may represent a potential alternative for the implementation of a novel insecticide.^18^ The advent of computational tools in recent years has facilitated the rapid integration of molecular modeling into the field of biological research.^19^

The hypothesis statement of this study is to employ the compounds of *S. longepedunculata* to inhibit the acetylcholinesterase activity of *Schizaphis graminum* neuronal transmission through the *in-silico* method.

## 2. Materials and methods

### 2.1. Securidaca longepedunculata (S. longepedunculata) *species*

*S. longepedunculata* Fresen, the species correct botanical name was confirmed by the International Plant Name Index (www.ipni.org).They belong to the Polygalaceae family and grow from 6 to 12 m tall. The sweetly scented pink to violet flowers are produced in early summer. The species is widely distributed throughout tropical Africa.^20^

### 2.2. Screening of secondary metabolites

The selection of bioactive compounds was based on the available literature data from the GC-MS analysis of *S. longepedunculata* extracts, as reported by.^21,21–23^ Consequently, data from sources such as Scopus, Google Scholar, and PubMed were sought that were less than 5-years old. The search term used on the aforementioned databases was “GC-MS analysis of *S. longepedunculata*.”

### 2.3. Ligand preparation

The 3D crystal structures of the molecules corresponding to the screening ligands were obtained from PubChem (https://pubchem.ncbi.nlm.nih.gov/). These screening bioactive compounds have all been optimized. All molecules retain the specified chirality, generate tautomers, and produce the corresponding ionized state at pH 7.0 ± 2.0 using Epik (Empirical pKa Prediction).^24^ The co-crystalline compound undergoes the same treatment.^25^

### 2.4. Protein preparation

The 3D crystal structure of the enzymatic protein complex was extracted from PDB (https://www.rcsb.org/). The enzyme complex was prepared using Schrodinger Maestro 2022 software. Initially, the water molecules and other cofactors were removed. Subsequently, the crystal structure’s hydrogen atoms and amino acid residues were incorporated into the system via Schrodinger’s “Protein Preparation Wizard.”^26^ At pH 7.4, the protonated and tautomeric amino acids were adjusted to the corresponding state. Ultimately, the energy of the hydrogen atom in the crystal structure was minimized using the OPLS 2005 atomic force field, and the heavy atom converged to an RMSD (root mean square deviation) of 0.3 Å.^27^

### 2.5. Molecular docking studies

Molecular docking studies were conducted to ascertain the inhibitory potential of secondary metabolites derived from GC-MS (gas chromatography/mass spectrometry) analyses of *S.* *longepedunculata* on acetylcholinesterase. The crystal structure of the protein, with the PDB (Protein Data Bank) identifier, was prepared, as was the ligand. Molecular clustering was conducted using tools such as Autodock Vina and Autodock Tools. The docked complex molecules were visualized using Biovia Discovery Studio version 2023. Docking and docking simulation were conducted using the Schrödinger Glide SP module.^28,29^ The resolution was 2.3 Å, and genetic algorithms were employed as a means of optimizing the process. The genetic algorithm routine is executed recursively, and after a reasonable number of cycles of searching and evaluating conformations.^30^

### 2.6. Molecular dynamics simulation (MDS) analysis

In order to better assess ligand-protein binding, molecular simulation was performed between phytocomponents with the lowest acetylcholinesterase-binding energies. The Desmond module (Schrödinger release 2022–4) was used to analyze the simulation. All simulations were run in replicate and the results are reported as an average of the three simulations to increase the credibility of the analysis. To ensure that the results were representative of the physiological environment, a solvation box was constructed at the 10 Å periodic boundary of the TIP3P water molecules.^31,32^ This box was then associated with the complex.^33^ The systems were neutralized with Na+ and Cl- counterions. Finally, the systems were minimized for 20 fs to avoid energy shocks. The solvated systems were equilibrated at 310 K as before final production. To maintain system stability, three additional equilibrations were performed at 200 K, 250 K, and 300 K, respectively. All the additional equilibrations are performed just before the final production at 310 K. The stabilized systems were simulated for 50 ns in the production cycle. The dynamic molecular basic algorithm was used for a remarkable improvement.^34^ The simulations are run in triplicate, and the results are reported as average of the three simulations. Molecular dynamics (MD) trajectories were stored at two ps intervals and analyzed using CPPTRAJ^35^ and the Bio3D package^36^ of the R program. The MD simulation trajectories were examined via simulation interaction diagrams using root mean square deviation (RMSD), root mean square fluctuation (RMSF), radius of gyration (Rg), and hydrogen bond analysis.^37^

In addition, the MMGBSA module of the AMBER21 tools was used to calculate bond-free energies.

### 2.7. Calcul MM/GBSA

To ascertain the stability of the ligand–protein complex generated by the docking stimulation, MM/GBSA (Molecular Mechanics/Generalized Born Surface Area) an approach was undertaken.^25^ The calculation of the binding free energy is based on the following formulae:

(a) $${\mathrm{\Delta G}}_{\mathrm{bind}}={\mathrm{\Delta G}}_{\mathrm{complex}}\;-\left({\mathrm{\Delta G}}_{\mathrm{protein}}+{\mathrm{\Delta G}}_{\mathrm{ligand}}\right)$$

(b) $${\mathrm{\Delta G}}_{\mathrm{bind}}=\mathrm{\Delta H}-\left({\mathrm{\Delta G}}_{\mathrm{sdvation}}+{\mathrm{T\Delta}}_{S}\right)$$

(c) $${\mathrm{\Delta G}}_{\mathrm{bind}}=\Delta E_{\mathrm{MM}}+\Delta G_{\mathrm{GB}}+{\mathrm{\Delta G}}_{\mathrm{SA}}-{\mathrm{T\Delta}}_{S}$$

ΔG_*bind*_ is binding energy; ΔG_*complex*_ is the free energy of a complex system;

ΔG_*protein*_ et ΔG_*ligand*_ is the free energy of the protein and ligand in the complex system; ΔG_*GB*_ et ΔG_SA_ is the contribution of polarity and non-polarity to the free energy of the solvent in the solvent environment.

ΔS represents the change in the entropy of the ligand structure during sampling.

ΔEMM is the gas-phase free energy. The parameters in MM/GBSA are the default: the solvation model is VSGB, and the sampling method is minimized. During the calculation, check “use constraints on flexible residues” to define residues around the receptor pocket as a flexible conformation.

## 3. Results

### 3.1. Molecular docking

The aim of this study was to evaluate phytochemical compounds with inhibitory activity on a neuronal transmission protein of *Schizaphis graminum*, acetylcholinesterase. A total of 178 secondary metabolites were collected from the GC-MS analysis of *Securidaca longepedunculata* (*S. longepedunculata)* available in the literature and less than 5-years old. On the basis of score docking, eight (8) bioactive compounds were selected (Table 1). The bioactive compounds (echimidine and populin) with the highest docking and score were subjected to molecular dynamics and free energy simulations.

**Table 1.** Docking score (Kj/mol) between candidate compounds and the 6ARX protein.

Compounds	Cas ID	Name	Docking Score
			6ARX
C1	5281729	Echimidine	−12.684
C2	92735	Populin	−11.863
C3	159278	Salidroside	−10.900
C4	91144	Farrerol	−8.703
C5	71585044	(3R)-5,7-Dihydroxy-6-methyl-3(4‘hydroxybenzyl) chroman-4-one	−7.702
C6	5281857	Geranylhydroquinone	−7.165
C7	16077444	1,7-dihydroxy-2,8-dimethoxyxanthone	−6.869
C8	5281126	Punicic acid	−6.501

### 3.2. Anchoring study of certain natural compounds with acetylcholinesterase, a basic aphid protease

**Figure 1.**
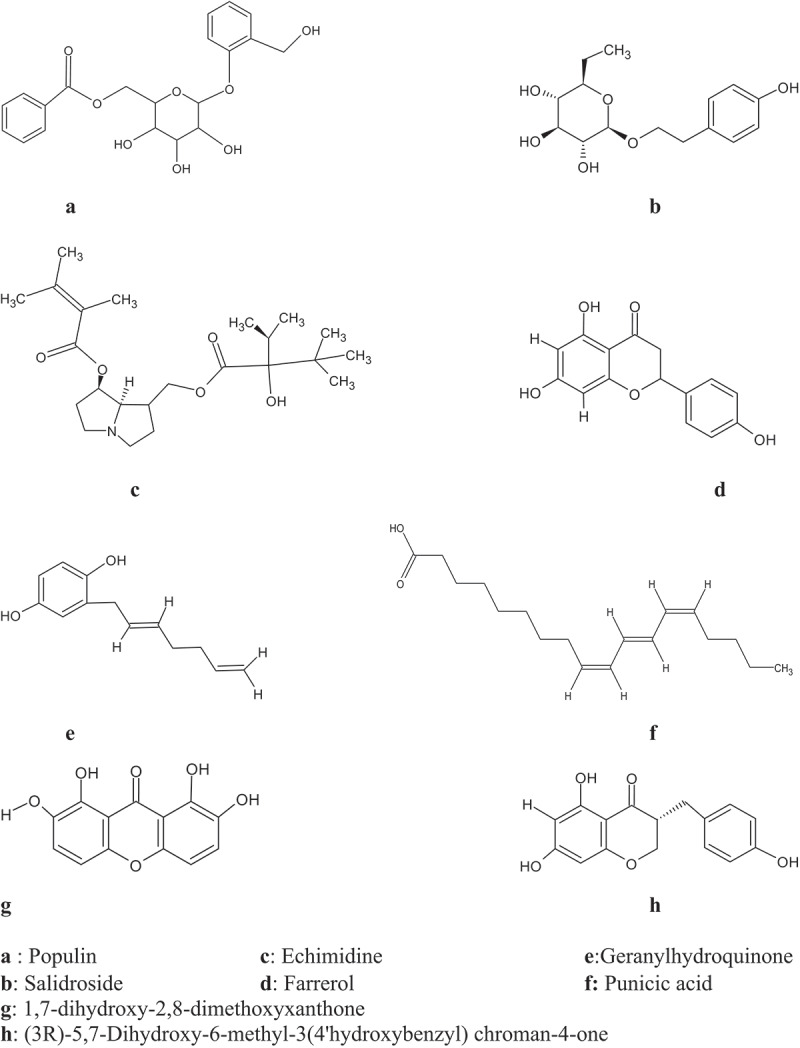
Different pharmacophores structure.

**Figure 2.**
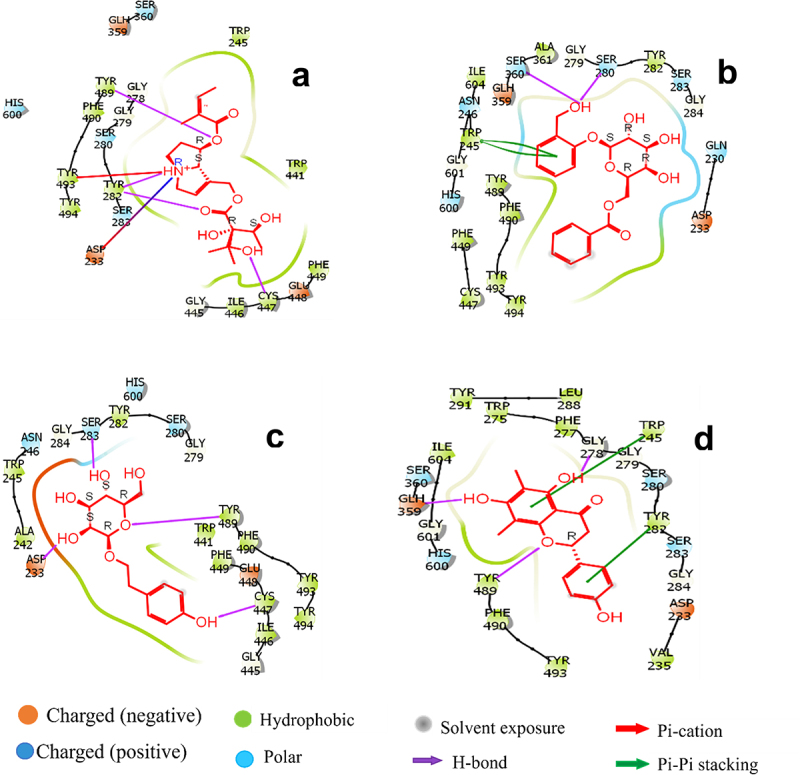
(a) Binding-interaction analysis of Echimidine at the binding site of 6ARX. (b) Binding-interaction analysis of populin at the binding site of 6ARX. (c) Binding-interaction analysis of Salidroside at the binding site of 6ARX. (d) Binding-interaction analysis of Farrerol at the binding site of 6ARX.

The results indicate that of the 21 residues displayed by the protein, only a few exhibited a greater affinity for specific ligands (Figure 1). Echimidine (Figure 2a) is well positioned in the protein’s active site, with the polar amino acids Ser 360; Ser 280; His 600; Ser 283; the charged Asp 233; Gln 359; Gln 448; the hydrophobic Tyr 489; Tyr 493; Tyr 282; Tyr 494; Trp 441; Phe 490; Ile 446; Cys 447; Trp 245, with a score of −12,684 kJ/mol. The hydroxyl group was engaged in a hydrogen bond with Cys 447, while the nitrophilic group was also involved in a hydrogen bond.

For the ligand, populin (Figure 2b) is correctly positioned in the catalytic site of acetylcholinesterase with polar residues Gln 230; Ser 280; Ser 283; Ser 360; Asn246; His 600; charged Asp 233; Gln 359; hydrophobic Ala 361; Phe 490; Ile 604; Trp 245; Tyr 489; Phe 449; Tyr 493; Tyr 282; Tyr 494; Cys 447, with a score of −11.863 Kj/mol. The hydroxyl group is engaged in a hydrogen bond with Ser 280 and Ser 360, and forms a Pi–Pi stack with Trp 245.

The ligand salidroside (Figure 2c) is well positioned in the catalytic site of the protein, with polar residues Ser 280; Ser 283; Asn 246; His 600; charged Asn 233; Glu 448; hydrophobes Tyr 282; Trp 245; Ala 242; Phe 490; Phe 449; Trp 441; Tyr 489; Tyr 493; Tyr 494; Cys 447; Ile 446, with a score of −10.90 kcal/mol. The hydroxyl group is linked to Ser 283, Cys 447, and Asp 233 via a hydrogen bond, as is Tyr 489 via the oxygen group.

Farrerol (Figure 2d) is positioned in the protein’s active site by the polar amino acids Ser 360; Ser 280; Ser 283; His 600; Asp 233; Gln 359; Val 235; Tyr 282; Trp 245; Phe 277; Leu 288; Trp 275; Tyr 291; Ile 604; Tyr 489; Phe 490; Tyr 493 with a score of −8,703 kcal/mol. The residues Gln 359 and Gly 275 are hydrogen-bonded to Farrerol via the hydroxyl group and to Tyr 489 via the oxygen group and a pi–pi stack with Trp 245 and Tyr 282.

### 3.3. Molecular dynamics simulation (MDS)

Molecular dynamics simulation will allow us to assess the stability of the ligands in the active site of the protein and possibly the mode of binding, the formation of hydrogen bonds and the interactions between the structures. The following parameters will be investigated: RMSD, RMSF, Rg, and free energies of binding with MMGBSA. Work will continue mainly with two ligands (echimidine and populin), on the basis of previous results but also to facilitate understanding.

**Figure 3.**
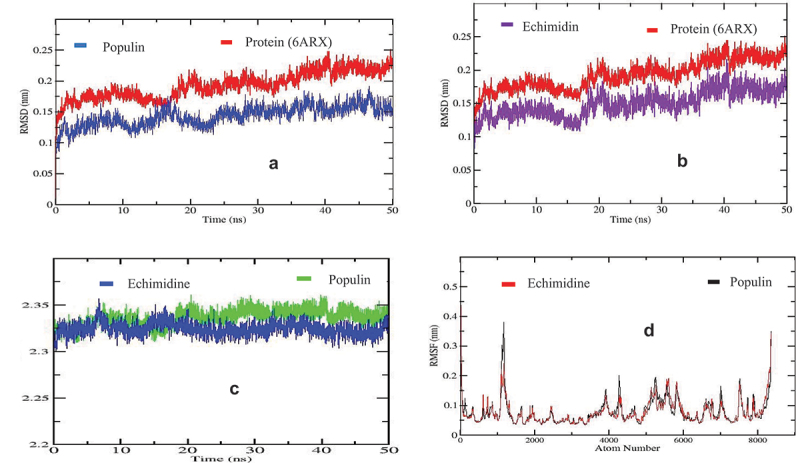
(a) RMSD analysis between populin and site of protein (6ARX). (b) RMSD analysis between echimidine and site of protein (6ARX). (c) Rg analysis between echimidine and populin. (d) RMSF analysis between echimidine and populin.

The RMSD of the main protein inhibitors shows that the interaction between populin and acetylcholinesterase (Figure 3a) and between echimidine and acetylcholinesterase (Figure 3b) shows slight fluctuations over the 50 ns period, with an RMSD value between 0.1 and 0.25 nm. Initially, between 0 and 10 ns, there was a slight RMSD deviation of 0.10 to 0.15 nm for populin. This was followed by a sawtooth trend from 0.15 to 0.25 ns, after which the RMSD stabilized for the remainder of the time (30–50 ns). Echimidine showed a similar trend with a slight deviation of 0.10–0.20 ns. In addition, the RMSD increased from 0.1 to 0.18 nm.

The radius of rotation (Rg) observed between populin and echimidine (Figure 3c) shows a transient variation in Rg during the 50 ns, with Rg values varying between 2.32 and 2.36 nm. Both complexes show a sharp increase during the first 8 ns, followed by a decrease from 2.35 to 2.30 nm between 9 and 15 ns. Thereafter, both structures show stability from 20 ns to the end of simulation, with the Rg value of populin being relatively lower than that of echimidine. This low value suggests tight folding due to energetic interactions and conformational entropy between the compounds.^38^ The RMSF fluctuation was initially low, then increased from 0.14 nm to 0.3 nm before returning to its normal value of 0.15 nm, reflecting the stability of the complex (Figure 3d). These high RMSF values indicate a period of low complex stability. In contrast, the majority of cases where these values are relatively low indicate the stability of the complex.

### 3.4. Calcul MM/GBSA

The analysis aimed to determine the affinity link between two major ligand-protein compounds and the key protein acetylcholinesterase. This affinity link was assessed by calculating the binding free energy concerning MM/GBSA for a 50 ns long MDS trajectory.

The binding energies calculated, the most important of which are the Van der Waals interaction energy ($\Delta E_{\mathrm{VDW}}$), Coulombic or electrostatic interaction energy ($\Delta E_{\mathrm{EL}}$), electrostatic contribution to the solvation-free energy ($\Delta E_{\mathrm{GB}}$), solvent-accessible surface area ($\Delta E_{\mathrm{Surf}}$), solvation-free energy ($\Delta E_{\mathrm{Solv}}$) (Table 2). The analysis demonstrated that echimidine exhibited a considerable degree of energy contribution $\Delta E_{\mathrm{GAS}}$ (−272.36 Kj/mol) et $\Delta E_{\mathrm{GB}}$ (243.55 Kj/mol) compared with populin, which was $\Delta E_{\mathrm{GAS}}$ (−161 Kj/mol) et de $\Delta E_{\mathrm{GB}}$ (133.09 Kj/mol). The release of these energies prompts the complex to maintain its stable conformation. Furthermore, echimidine has been shown to possess a high electrostatic energy $\Delta E_{\mathrm{EL}}$ (−232.07 Kj/mol) in comparison to populin, which is recorded at −119.38 Kj/mol. This illustrates the number of electrons necessary to reinforce the diverse bonds between echimidine and the protein, thus ensuring its stability within the active site.

**Table 2.** Binding free energy (Kj/mol) calculation of complex populin and echimidine determined by MMGBSA.

Interactions	6ARX-Echimidine	6ARX -Populin
$\Delta E_{\mathrm{VDW}}$	−40.29 ± 0.05	−41.72 ± 0.04
$\Delta E_{\mathrm{EL}}$	−232.07 ± 0.24	−119.28 ± 0.08
$\Delta E_{\mathrm{GB}}$	243.55 ± 0.21	133.09 ± 0.06
$\Delta E_{\mathrm{Surf}}$	−5.80 ±0.01	−6.19 ± 0.00
$\Delta E_{\mathrm{GAS}}$	−272.36 ±0.24	−161.00 ± 0.09
$\Delta E_{\mathrm{Solv}}$	237.75 ±0.21	126.90 ± 0.06

$\Delta E_{\mathrm{VDW}}$= van der Waals contribution from MM; $\Delta E_{\mathrm{EL}}$= electrostatic energy as calculated by the MM force field; $\Delta E_{\mathrm{GB}}$= the electrostatic contribution to the solvation-free energy calculated by GB; $\Delta E_{\mathrm{Surf}}$= solvent-accessible surface area; $\Delta E_{\mathrm{GAS}}$_=_ gas-phase interaction energy; $\Delta E_{\mathrm{Solv}}$_=_ solvation-free energy.

## 4. Discusion

Acetylcholinesterase is a fundamental protein involved in the neuronal transmission of excitatory messages. Inactivation of this enzyme leads to paralysis and, in extreme cases, death. Biological control of this wheat pest has involved computerized simulation of *Securidaca longepedunculata* (*S. longepedunculata)* bioactives, which have been used to inhibit the hydrolyzing activity of the enzyme from disrupting excitatory transmission in the aphid’s cholinergic synapses. Some of the *S. longepedunculata* ligands, such as echimidine and populin, showed high affinity and stability within the active site of the protein. A series of activities including molecular docking, molecular dynamics simulations, and bond-free energy calculations were carried out to gain a deeper understanding of the properties of these various inhibitory molecules.^39,40^ Some of these compounds showed the highest binding energies to the protein, as exemplified by echimidine, populin, salidroside, and farrerol (Table 1). They also showed strong anchoring in the active site of the protein by binding to the amino acids. Echimidine, with an anchoring score of −12.684 Kj/mol, showed a stronger binding affinity to residues Tyr489, Tyr282 and Cys447 via the carbonyl and hydroxyl groups, forming robust hydrogen bonds. In addition to the carbonyl group, it showed a p–cation interaction with Tyr493 (Figure 2a). In contrast, populin was strongly bound to residues Ser360 and Ser280 through its aromatic ring, forming a stable hydrogen bond with acetylcholinesterase. It was found to have a p–p interaction with Trp245 (Figure 2b).

For RMSD, echimidine showed conformational stability of the protein compared to populin, salidroside, and farrerol (Figure 3). This stability is probably due to the hydrogen bonds formed between the ligand and the acetylcholinesterase residues. Indeed, the amino acids of the protein can choose a type of binding with a ligand to ensure its stable conformation.^41^ Amino acid regions of the active site that are exposed to solvent are more rigid, while those on the sides that are not exposed to solvent are more unstable and capable of fluctuating conformations.^42^ This instability can be periodic and localized during molecular simulation, stabilizing the conformation to a normal order (Figure 3a,b). During molecular dynamics simulation, amino acids attempt to form new bonds with the ligand to stabilize themselves, causing fluctuations in their conformations.^43^ This conformational behavior of the different amino acids is consistent with their fluctuations.^44^ The results indicate that the complexes underwent less fluctuations during molecular dynamics simulation (Figure 3d) due to the conformational stability (Figure 3c), likely due to the strong bonds formed with the residues. The RMSD of echimidine, followed by its RMSF, indicates that it is more stable than populin in the catalytic phase of acetylcholinesterase. The stability observed during molecular dynamics simulation can be attributed to two factors: the formation of specific bonds between the acetylcholinesterase amino acids and echimidine, and the presence of van der Waals interactions, which in this case are particularly strong compared to populin (Table 2). Calculating the binding energy (MMGBSA) in ligand-protein complexes also allowed us to understand the affinity of ligands in catalytic sites. The higher the binding energy, such as the van der Waals electrostatic energy (Table 2), the more stable the complex. In line with previous findings, echimidine exhibited the highest energies compared to populin. These emitted energies facilitated the maintenance of pre-established bonds during simulation. Indeed, during molecular dynamics simulation, the protein optimizes its conformation to better adapt to the catalytic site of the receptor.^40^

Previous results indicate that echimidine and populin can form continuous and stable interactions with amino acids throughout the course of molecular dynamics simulation. Therefore, it was essential to provide amino acids with considerable free energy to maintain this state.

## 5. Conclusion

In this study, we used bioactive compounds from *Securidaca longepedunculata (S. longepedunculata)* to inhibit the activity of acetylcholinesterase (AChE), an enzyme essential for the transmission of excitatory messages from cholinergic receptors in neuronal synapses. Docking activity was used to select eight phytocompounds with high anchoring scores. Two of the eight (08) candidate compounds, namely echimidine and populin, were considered more promising based on their docking and anchoring scores and were further analyzed. Based on the simulation of molecular dynamics and the contribution of energy to stability and conformational fluctuations, echimidine and populin were the most convincing in terms of protein affinity and stability. Both candidates showed remarkable stability in the catalytic site of acetylcholinesterase, forming strong hydrogen bonds with key amino acids. The free energy (MMGBSA) of the protein was calculated and showed that echimidine and populin could bind stably to the binding pocket and form strong, continuous hydrogen bonds with amino acids. However, further studies are needed to evaluate the efficacy of these two compounds *in vitro* and *in vivo* to improve the effectiveness of the biopesticide.

## Data Availability

Data is contained within the article.
